# Fluorescence in situ hybridization as prognostic predictor of tumor recurrence during treatment with Bacillus Calmette–Guérin therapy for intermediate- and high-risk non-muscle-invasive bladder cancer

**DOI:** 10.1007/s12032-017-1033-z

**Published:** 2017-09-02

**Authors:** Esmee I. M. L. Liem, Joyce Baard, Evelyne C. C. Cauberg, Mieke T. J. Bus, D. Martijn de Bruin, M. Pilar Laguna Pes, Jean J. M. C. H. de la Rosette, Theo M. de Reijke

**Affiliations:** 1 Present Address: Department of Urology, Academic Medical Center, Meibergdreef 9, 1105 AZ Amsterdam, The Netherlands; 2 Department of Biomedical Engineering and Physics, Academic Medical Center, Meibergdreef 9, 1105 AZ Amsterdam, The Netherlands

**Keywords:** Bladder cancer, Non-muscle-invasive bladder carcinoma (NMIBC), Bacillus Calmette–Guérin (BCG), Fluorescence in situ hybridization (FISH), Biomarker, Recurrence

## Abstract

A significant number of patients with intermediate- or high-risk bladder cancer treated with intravesical Bacillus Calmette–Guérin (BCG) immunotherapy are non-responders to this treatment. Since we cannot predict in which patients BCG therapy will fail, markers for responders are needed. UroVysion^®^ is a multitarget fluorescence in situ hybridization (FISH) test for bladder cancer detection. The aim of this study was to evaluate whether FISH can be used to early identify recurrence during treatment with BCG. In a multicenter, prospective study, three bladder washouts at different time points during treatment (*t*_0_ = week 0, pre-BCG, *t*_1_ = 6 weeks following TURB, *t*_2_ = 3 months following TURB) were collected for FISH from patients with bladder cancer treated with BCG between 2008 and 2013. Data on bladder cancer recurrence and duration of BCG maintenance therapy were recorded. Thirty-six (31.6%) out of 114 patients developed a recurrence after a median of 6 months (range 2–32). No significant association was found between a positive FISH test at *t*_0_ or *t*_1_ and risk of recurrence (*p* = 0.79 and *p* = 0.29). A positive *t*_2_ FISH test was associated with a higher risk of recurrence (*p* = 0.001). Patients with a positive FISH test 3 months following TURB had a 4.0–4.6 times greater risk of developing a recurrence compared to patients with a negative FISH. Patients with a positive FISH test 3 months following TURB and induction BCG therapy have a higher risk of developing tumor recurrence. FISH can therefore be a useful additional tool for physicians when determining a treatment strategy.

**Electronic supplementary material:**

The online version of this article (doi:10.1007/s12032-017-1033-z) contains supplementary material, which is available to authorized users.

## Introduction

Non-muscle-invasive bladder cancer (NMIBC) is a heterogeneous histopathological condition with different prognoses. Based on risk factors, patients are classified into risk groups with low, intermediate and high risk of recurrence and progression [1]. For intermediate- or high-risk NMIBC, adjuvant intravesical therapy with Bacillus Calmette–Guérin (BCG) is recommended in the guidelines of the European Association of Urology and the American Urological Association [1, 2].

In spite of its effectiveness, intravesical BCG therapy is not devoid of limitations [3]. BCG treatment may induce local side effects in 62.8% and systemic side effects in 30.6% of patients with possible fatal outcome [4]. This may lead to interruption or discontinuation of BCG treatment in up to 20% of patients [5]. Besides, in up to 40% of patients BCG treatment fails [6–8]. BCG failure can be divided into different types: BCG intolerant, refractory and relapsing. BCG intolerant patients have to stop due to side effects, whereas BCG refractory patients do not respond to BCG induction therapy and have persistent disease, while BCG relapsing patients initially do respond to BCG treatment, but after a disease-free period develops a recurrence [9, 10]. Since BCG is mainly given to treat patients with a high risk of progression to muscle-invasive disease, it is important to identify non-responding patients early. However, currently no diagnostic tool is available to discriminate between BCG responders and BCG non-responders. A predictive test is desirable and might be helpful in treatment decision.

UroVysion^®^ (Abbott Molecular, Illinois, USA) FISH is able to detect genetic alterations most commonly associated with bladder cancer. The assay detects aneuploidy of chromosomes 3, 7 and 17 and a deletion of locus 9p21 [11]. Since FISH is based on detection of genetic alterations, results or interpretation of the test will not be influenced by the inflammatory response of the bladder to BCG, as opposed to cystoscopy and urine cytology [12].

If we can predict which patients are at risk of developing a recurrence during BCG treatment, it is possible to prevent under-treatment by timely changing from BCG therapy to other intravesical therapy or to radical therapy, i.e., radical cystectomy. Furthermore, early identification of BCG non-responders will limit the associated risks of BCG therapy. The aim of this study is to determine the usefulness of FISH as predictor of tumor recurrence in patients with NMIBC treated with BCG instillations.

## Materials and methods

### Patient inclusion

From 2008 to 2013, five centers included patients with NMIBC treated with BCG instillations in a prospective study evaluating the accuracy of FISH in bladder washout. Informed consent was verbally obtained of all participants prior to inclusion. Patients had histologically confirmed primary or recurrent intermediate- or high-risk NMIBC (CIS, Ta, T1, all grades) and were scheduled for BCG induction therapy after complete transurethral resection of the bladder tumor(s) (TURB). Administration of a single immediate postoperative chemotherapy instillation or re-resection was left to the discretion of the treating urologist. Exclusion criteria included presence of muscle-invasive disease, no histologic confirmation of bladder tumor and synchronous upper urinary tract urothelial carcinoma.

### BCG instillation protocol

All patients were scheduled to receive at least induction BCG therapy of six weekly instillations following TURB. Maintenance therapy was administered depending on hospitals’ protocols. In general, maintenance therapy consisted of three weekly instillations during 1 to 3 years (at 3, 6, 12, 18, 24, 30, 36 months). Patients were followed by cystoscopy every 3 months during the first two years after inclusion or until a recurrence was diagnosed. Data on bladder cancer recurrence and duration of BCG maintenance therapy were recorded. A recurrence was defined as histopathologically proven NMIBC or muscle-invasive disease (*T* ≥ 2). Tumor grade was assessed based on the 1973 WHO classification. Progression was defined as the histologic confirmation of muscle-invasive disease (*T* ≥ 2).

### Bladder washout protocol

Bladder washouts (BWOs) for FISH evaluation were collected at three time points: before the first BCG instillation (*t*_0_), before the last induction BCG instillation at 6 weeks (*t*_1_) and at 3 months during first cystoscopy follow-up (*t*_2_). BWOs at *t*_0_ and *t*_1_ were done via a catheter, and 50 cc 0.9% saline was used to flush the bladder. At *t*_2_, the BWO was done at the end of the cystoscopy via the working channel of the cystoscope. Each BWO was preserved in carbowax (polyethylene glycol). Cytospins were made within 72 h and stored in a −20 °C freezer until FISH test was performed.

### FISH protocol

All BWOs were analyzed using the multitarget UroVysion^®^ bladder cancer kit. The FISH kit is composed of a mixture of four-target multicolor probes, three chromosome enumeration probes (CEP 3, CEP 7 and CEP 17) and one single locus-specific indicator probe (LSI 9p21). Cytospins were made of collected BWOs and fixed using Carnoy’s solution (3:1 methanol/glacial acetic acid). Slides were pre-treated using the Vysis pre-treatment kit (Abbott Molecular, Illinois, USA), and FISH was performed according to the manufacturer’s instructions provided with the assay. In short, slides were denatured in 2 × SSC at 73 ± 1 °C for 2 min and incubated in pepsin buffer at 37 °C for 10 min. After 5-min washing at room temperature with phosphate-buffered saline (PBS), the slides were fixed in 1% formaldehyde during 5 min. The slides were washed again in PBS at room temperature for 5 min and dehydrated in consecutively 70, 85 and 100% ethanol, for 1 min each. For hybridization the multitarget UroVysion^®^ probe mixture was added and incubated overnight at 73 °C (denaturation 2 min) and 37 °C (hybridization 8–16 h) using the ThermoBrite System. Post-hybridization the slides were washed in 0.4 SSC at room temperature for 5 min, 0.4 SSC at 73 °C for maximum 2 min and 2 × SSC at room temperature for 1 min. Nuclei were counterstained with DAPI (4′,6-diamidino-2-phenylindole). FISH assays were examined using a fluorescence microscope (Leica DM 5000B and Leica DM 5500) with the following filters: A4 (DAPI), TX2 (CEP 3, red), L5 (CEP 7, green), SAQ (CEP 17, aqua) and SGO (LSI 9p21, gold).

### Data analysis

Slides were screened for 25 morphologically abnormal cells (large nuclear size, irregular nuclear shape, patchy DAPI staining or cell clusters) and considered positive if one of the following criteria were met: ≥4 cells have a gain of 2 or more chromosomes (3, 7 or 17), or ≥12 cells have a loss of both copies of LSI 9p21 [13]. During the course of the trial, three designated researchers evaluated all slides. The researchers were instructed and trained by one of the manufacturer’s cytogenetic consultants.

### Statistics

Data were analyzed using SPSS Statistics version 23. Descriptive statistics were used for patient characteristics. Patient and tumor characteristics of patients with a FISH result available at *t*_1_ and *t*_2_ were compared with the whole cohort with a FISH result available at *t*_0_, to evaluate whether missing cases at *t*_1_ and *t*_2_ influenced the results. *P* values were calculated by using one-sample test proportion. Kaplan–Meier method was used to estimate recurrence-free survival and progression-free survival based on positive or negative FISH test at the three time points (*t*_0_, *t*_1_ and *t*_2_). The log-rank test was used for statistical significance. Hazard ratios were calculated using Cox proportional regression analysis. Sensitivity, specificity, positive predictive value (PPV), negative predictive value (NPV) and accuracy of the test at each time point during evaluation were calculated using 2 × 2 tables.

## Results

### Patient characteristics and outcomes

In total 147 patients were enrolled during the study period with 114 patients finally being eligible for data evaluation. Patient and tumor characteristics are summarized in Tables 1 and 2. Sixty-six patients received BCG maintenance (4–25 months). Median follow-up for the whole cohort was 23 months (range 2–32). During follow-up 36 patients (31.6%) developed a recurrence (Table 3) at a median time of 6 months (range 3–28). Disease progression to muscle-invasive bladder cancer occurred in 4 of the 36 patients after a median time of 13 months (range 7–23). High-grade tumor recurrence occurred in 25 patients (Table 3). During follow-up, one patient developed a ureter tumor and 15 patients died: 3 patients as a result of metastatic bladder cancer, 6 patients due to non-urologic reasons and 6 patients with an unknown cause. Six patients were lost to follow-up, with no available data.

**Table 1:** Patient characteristics

	*n*	%
Patients (*n*)	114	
Male (*n*, %)	88	77.2%
Female (*n*, %)	26	22.8%
Mean age (years, range)	70.7	42–94
Median follow-up (months, range)	23	2–32
History of bladder cancer (*n*, %)	34	29.8%
Previous intravesical treatment (*n*, %)	15	13.2%
Mitomycin C	8	
BCG	7	

*BCG* Bacillus Calmette–Guérin

**Table 2:** Tumor characteristics

	*n*	%
Tumor stage		
CIS only	23	20.2
Ta	43	37.7
T1	48	42.1
Tumor grade		
CIS only	23	20.2
G1	6	5.3
G2	7	6.1
G2 + CIS	4	3.5
G3	57	50.0
G3 + CIS	17	14.9
Intermediate risk	7*	6.2
High risk	105*	93.8
Single tumors	42	36.8
Multifocal	72	63.2

*CIS* carcinoma in situ

* Two patients could not be classified, because information regarding tumor size was missing

**Table 3:** Recurrence during 24-month follow-up

	CIS only	G1	G2	G3	G3 + CIS	Total
CIS only	9	0	0	0	0	9
Ta	0	5	5	3	2	15
T1	0	0	1	6	1	8
T2	0	0	0	3	1	4
Total	9	5	6	12	4	36

### FISH results

**Fig. 1:**
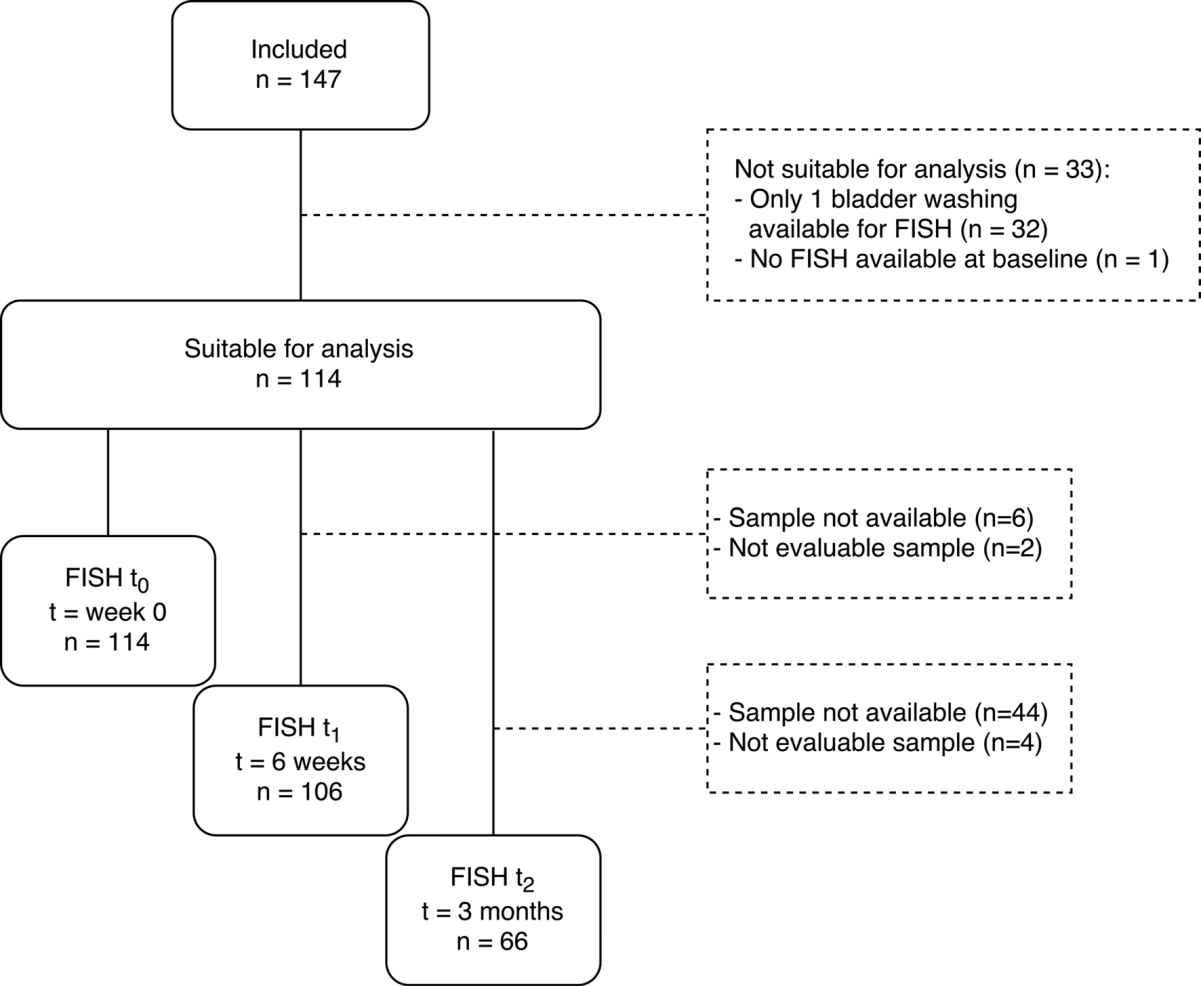
Flowchart showing patient inclusion and exclusion

Patients were considered suitable for analysis if at least two evaluable BWOs were available for FISH, with one sample being collected at *t*_0_ and a second sample at either *t*_1_ or *t*_2_ (Fig. 1). Of 58 patients (50.9%) FISH results at all 3 time points were available, and of 56 patients (49.1%) 2 FISH samples were available (*n* = 48 for *t*_0_ and *t*_1_, *n* = 8 for *t*_0_ and *t*_2_). FISH test was available at *t*_0_ in 114 patients and was positive in 48 patients (42.1%). At *t*_1_ FISH test was available in 106 patients. Thirty-six patients converted from pre-BCG-positive FISH to post-BCG-negative FISH at *t*_1_. In total 16 patients (15.1%) had a positive FISH result at *t*_1_. At first cystoscopic surveillance or *t*_2_, 66 FISH results were available, of which 18 were positive (27.3%). Of these patients, 10 patients (15.2%) initially had a negative pre-BCG FISH result that converted to a positive FISH result at *t*_2_.

### Survival analysis

**Fig. 2:**
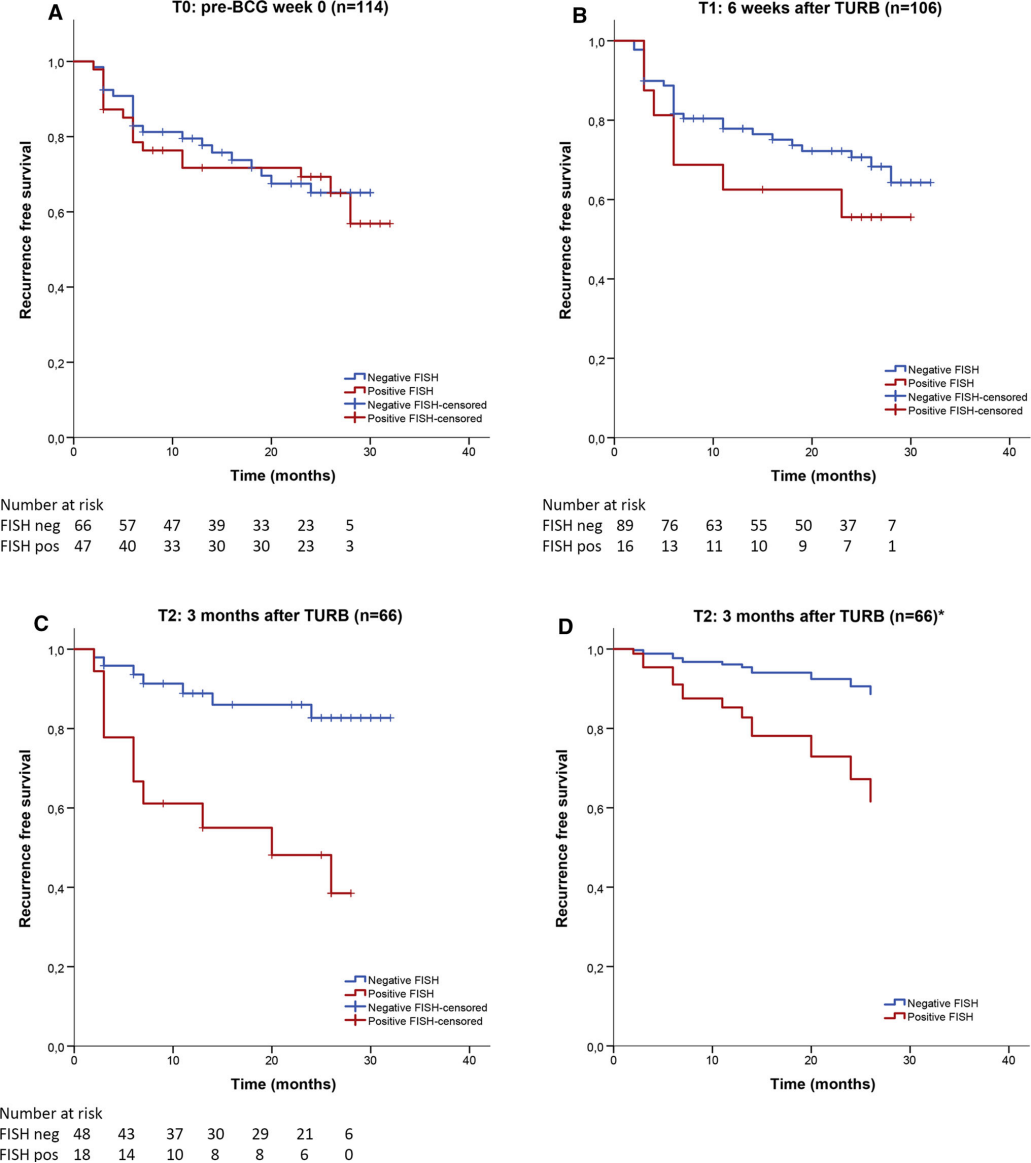
Recurrence-free survival curves of *t*_0_, *t*_1_ and *t*_2_ and hazard curve of *t*_2_, corrected for possible confounding. **a** Kaplan–Meier curve of patients with a positive FISH test versus negative FISH test pre-BCG (*t*_0_). **b** Kaplan–Meier curve of patients with a positive FISH test versus a negative FISH test post-BCG induction at 6 weeks (*t*_1_). **c** Kaplan–Meier curve of patients with a positive FISH test versus negative FISH test post-BCG at 3 months (*t*_2_). **d** Hazard curve of *t*_2_, corrected for immediate postoperative instillation, repeat TURB and number of BCG maintenance instillations

Kaplan–Meier curves for recurrence of the whole cohort and for the three time points in which FISH was performed are shown in Fig. 2. No association between a positive FISH result and tumor recurrence was found at *t*_0_ (*p* = 0.79), and a nonsignificant correlation was observed at *t*_1_ (*p* = 0.29). At *t*_2_ a positive FISH test was significantly associated with a higher risk of recurrence (*p* = 0.001). Cox regression showed that patients with a positive FISH test at *t*_2_ had a 4.6 times greater risk of tumor recurrence compared to patients with a negative FISH test at 3 months following TURB (95% CI: 1.71–11.84). When corrected for an immediate postoperative chemotherapy instillation, repeat TURB and number of maintenance BCG instillations, a positive FISH test at *t*_2_ had a 4.0 greater risk (95% CI: 1.45–11.10) of tumor recurrence compared to patients with a negative FISH test.

Due to the small number of progression events during the study period, a separate Kaplan–Meier analysis for this outcome was not possible.

### Diagnostic test evaluation

Sensitivity of FISH at *t*_0_, *t*_1_ and *t*_2_ was 44, 21 and 59%, and specificity was 59, 88 and 84%, respectively. For the three different points in time, PPV was 33, 44 and 56% and NPV was 70, 71 and 85%, respectively. Accuracy of the FISH test at *t*_0_, *t*_1_ and *t*_2_ was 54, 67 and 77%, respectively (Table 4).

**Table 4:** Evaluation of UroVysion^®^ FISH at *t*_0_, *t*_1_ and *t*_2_

	*T*_0_			
	Recurrence during FU			Sens = 0.44 (16/36)
FISH at *t*_0_	Yes	No	Total	Spec = 0.59 (46/78)
Positive	16	32	48	PPV = 0.33 (16/48)
Negative	20	46	66	NPV = 0.70 (46/66)
Total	36	78	114	Acc = 0.54 ((16 + 46)/114)

	*T*_1_			
	Recurrence during FU			Sens = 0.21 (7/33)
FISH at *t*_1_	Yes	No	Total	Spec = 0.88 (64/73)
Positive	7	9	16	PPV = 0.44 (7/16)
Negative	26	64	90	NPV = 0.71 (64/90)
Total	33	73	106	Acc = 0.67 ((7 + 64)/106)

	*T*_2_			
	Recurrence during FU			Sens = 0.59 (10/17)
FISH at *t*_2_	Yes	No	Total	Spec = 0.84 (41/49)
Positive	10	8	18	PPV = 0.56 (10/18)
Negative	7	41	48	NPV = 0.85 (41/48)
Total	17	49	66	Acc = 0.77((10 + 41)/66)

*FU* follow-up, *Sens* sensitivity, *Spec* specificity, *PPV* positive predictive value, *NPV* negative predictive value, *Acc* accuracy

## Discussion

The results from this study confirm earlier data from smaller or single-center studies, establishing the potential of FISH as a part of a predictive diagnostic workup. This study demonstrates that the UroVysion^®^ FISH test 3 months following TURB and BCG induction can be of value when considering disease management for patients with intermediate- or high-risk NMIBC. Patients with a positive FISH test at t_2_ had a 4.0–4.6 times greater risk to develop a recurrence than patients with a negative FISH test. At *t*_2_, sensitivity, specificity and accuracy of FISH were 59, 84 and 77%, respectively. On the contrary, despite a trend at *t*_1_, the results of the FISH test at *t*_0_ and *t*_1_ were not significantly associated with the risk of tumor recurrence. Risk assessment for tumor progression using FISH could not be determined due to the small number of progression events.

The literature regarding the efficacy of UroVysion^®^ for predicting recurrence risk following adjuvant instillations is scarce. Kipp et al. and Whitson et al. published results of patients who received bladder instillations using intravesical therapy including BCG, MMC and Thiotepa [14, 15]. Both groups reported that a positive FISH test following intravesical therapy was associated with a higher risk of recurrence. Additionally, a positive FISH test prior to intravesical treatment was associated with a higher risk of recurrence, and a positive FISH test following intravesical treatment was associated with a higher risk of progression to muscle-invasive disease [14]. Three other studies focused on risk assessment for tumor recurrence using FISH in patients treated with BCG instillations only [16–18]. These groups also reported that a positive FISH test following BCG therapy was associated with a higher risk of recurrence. Our results at t_0_ and t_1_ are in line with results reported by Mengual et al. and Savic et al. [16, 17]. However, Kamat et al. found a positive association for *t*_0_. This discordance could be explained by the difference in patient cohorts. In the cohort evaluated by Kamat et al., 89% of the patients had a previously treated bladder tumor and 48% had CIS as secondary finding, whereas in our cohort this was 30 and 18%, respectively [18].

Although not significant, the association between a positive FISH test at t_1_ and the risk of recurrence indicates a positive trend. We hypothesize that patients with a false positive FISH at t_1_ did not fully benefit from the BCG induction therapy yet, since BCG-induced delayed immune reaction may differ in each patient [19, 20].

At first cystoscopic surveillance following TURB, 18 patients had a positive FISH test. However, some had a false positive FISH test. A follow-up of 2 years might be too short to detect progression and leads to underestimation of recurrent and progressive disease. Conversely, a negative FISH test 3 months following initial TURB does not exclude patients to develop a recurrence. In our study 7 patients had a false negative FISH test at *t*_2_ (15% of all patients with a negative FISH result at *t*_2_) and did develop a recurrence bladder tumor during follow-up at a median of 7 months (range 2–24). Of these, 2 patients progressed to muscle-invasive disease (supplemental table S1). Although UroVysion^®^ is designed to detect genetic changes associated with most bladder cancers, some bladder tumors have different genetic changes that will not be detected using this test [21–25].

A limitation of this study is the number of BWO samples not available or suitable for analysis. This reduces the power of the study. Secondly, the number of patients with an available FISH result at *t*_2_ is limited. When comparing patients with available FISH results at *t*_2_ and at *t*_0_, patient and tumor characteristics were similar, except for tumor focality. This could imply that tumors of patients that had a FISH result available at *t*_2_ were slightly more aggressive (supplemental tables S2 and S3). Furthermore, in this study BWOs were used for logistic reasons. Though UroVysion^®^ is intended to be performed in voided urine samples, it has been demonstrated that the test is valid when performed in BWO samples [13]. Also, BWOs were processed over the course of 7 years. It cannot be ruled out that during this period some samples were improperly handled or stored. However, six and a half years after collecting the urine samples still good fluorescent signals were obtained. Lastly, the duration of BCG maintenance therapy is still a subject of debate. Patients received a 6-week induction course of BCG, and in the majority of cases this was followed by BCG maintenance therapy depending on hospital protocol. This may have influenced the chance of developing a recurrence [26]. We could not assess the effect of the different maintenance protocols.

Based on our results, a positive UroVysion^®^ FISH result alone is not sufficient to decide to switch from BCG to radical cystectomy at an early stage (3 months following TURB). There is a substantial risk of overtreatment if all patients with a positive FISH test at *t*_2_ would undergo more aggressive treatment. A positive FISH test following BCG treatment (*t*_2_) is, however, associated with a higher risk of developing a recurrence. A recent update of the guideline of the American Urological Association recommends the use of UroVysion^®^ to assess response to intravesical BCG therapy (level of recommendation: expert opinion) [2]. We recommend for future clinical trials to incorporate FISH at later time points after induction therapy (≥3 months following initial TURB).

## Conclusion

This study demonstrates that a positive UroVysion^®^ test at 3 months following TURB and induction BCG therapy for intermediate- and high-risk urothelial carcinoma of the bladder is associated with a statistical significant higher risk of recurrence. Therefore, it can be a useful tool for urologists to assess which patients have a higher risk of developing a recurrence.

## Electronic supplementary material

Below is the link to the electronic supplementary material.

Supplementary material 1 (DOCX 33 kb)

Supplementary material 2 (DOCX 35 kb)

Supplementary material 3 (DOCX 36 kb)
