# Supplementary material for: Fluorescence in situ hybridization as prognostic predictor of tumor recurrence during treatment with Bacillus Calmette–Guérin therapy for intermediate- and high-risk non-muscle-invasive bladder cancer
Source: Med Oncol. 2017 Sep 2;34(10):172. doi: 10.1007/s12032-017-1033-z (PMC5581817; doi:10.1007/s12032-017-1033-z)
Supplement: Supplementary file 1 — Supplementary material 1 (DOCX 33 kb) [file 12032_2017_1033_MOESM1_ESM.docx]

Supplemental table 1 Tumor characteristics of recurrence with a false negative FISH test at t_2_

|  | CIS only | G2 | G3 | G3 + CIS | Total |
| --- | --- | --- | --- | --- | --- |
| CIS only | 2 | 0 | 0 | 0 | 2 |
| Ta | 0 | 2 | 1 | 0 | 3 |
| T2 | 0 | 0 | 1 | 1 | 2 |
| Total | 2 | 2 | 2 | 1 | 7 |
