# Supplementary material for: Fluorescence in situ hybridization as prognostic predictor of tumor recurrence during treatment with Bacillus Calmette–Guérin therapy for intermediate- and high-risk non-muscle-invasive bladder cancer
Source: Med Oncol. 2017 Sep 2;34(10):172. doi: 10.1007/s12032-017-1033-z (PMC5581817; doi:10.1007/s12032-017-1033-z)
Supplement: Supplementary file 2 — Supplementary material 2 (DOCX 35 kb) [file 12032_2017_1033_MOESM2_ESM.docx]

**Supplemental table 2** Patient characteristics (t_0_, t_1_, t_2_)

|  | T_0_ | T_1_ | T_2_ | P (t_0_ vs. t_2_) |
| --- | --- | --- | --- | --- |
| Patients (n) | 114 | 106 | 66 |  |
| Male (n, %) | 88 (77.2%) | 81 (76.4%) | 51 (77.3%) | 0.548 |
| Female (n, %) | 26 (22.8%) | 25 (23.6%) | 15 (22.7%) |  |
| Mean age (years, range) | 70.7 (42-94) | 70.5 (42-94) | 72.3 (50-94) |  |
| Median follow-up (months, range) | 23 (2-32) | 24 (2-32) | 23 (2-32) |  |
| History of bladder cancer (n, %) | 34 (29.8%) | 32 (30.2%) | 19 (28.8%) | 0.467 |
| Previous intravesical treatment (n, %) | 15 (13.2%) | 13 (12.3%) | 10 (15.2%) |  |
| Mitomycin C | 8 | 8 | 5 |  |
| BCG | 7 | 5 | 5 |  |
| BCG = Bacillus Calmette-Guérin | |  |  |  |
