# Supplementary material for: Fluorescence in situ hybridization as prognostic predictor of tumor recurrence during treatment with Bacillus Calmette–Guérin therapy for intermediate- and high-risk non-muscle-invasive bladder cancer
Source: Med Oncol. 2017 Sep 2;34(10):172. doi: 10.1007/s12032-017-1033-z (PMC5581817; doi:10.1007/s12032-017-1033-z)
Supplement: Supplementary file 3 — Supplementary material 3 (DOCX 36 kb) [file 12032_2017_1033_MOESM3_ESM.docx]

**Supplemental table 3** Tumor characteristics (t_0_, t_1_, t_2_)

|  | T_0_ | T_1_ | T_2_ | P (t_0_ vs t_2_) |
| --- | --- | --- | --- | --- |
| Tumor stage (n, %) |  |  |  |  |
| CIS only | 23 (20.2%) | 21 (19.8%) | 14 (21.2%) | 0.467 |
| Ta | 43 (37.7%) | 39 (36.8%) | 26 (39.4%) | 0.434 |
| T1 | 48 (42.1%) | 46 (43.4%) | 26 (39.4%) | 0.377 |
| Tumor grade |  |  |  |  |
| CIS only | 23 (20.2%) | 21 (19.8%) | 14 (21.2%) | 0.467 |
| G1 | 6 (5.3%) | 6 (5.7%) | 3 (4.5%) | 0.534 |
| G2 | 7 (6.1%) | 7 (6.6%) | 3 (4.5%) | 0.422 |
| G2 + CIS | 4 (3.5%) | 4 (3.8%) | 2 (3.0%) | 0.592 |
| G3 | 57 (50.0%) | 51 (48.1%) | 34 (51.6%) | 0.902 |
| G3 + CIS | 17 (14.9%) | 17 (16.0%) | 10 (15.2%) | 0.530 |
| Intermediate-Risk | 7* (6.2%) | 7* (6.7%) | 2** (3.1%) |  |
| High-Risk | 105* (93.8%) | 97*(93.3%) | 63**(96.9) | 0.224 |
| Single tumors | 42 (36.8%) | 39 (36.8%) | 17 (25.8%) |  |
| Multifocal | 72 (63.2%) | 67 (63.2%) | 49 (74.2%) | 0.039 |
| CIS = Carcinoma In Situ  * 2 patients could not be classified because information regarding tumor size was missing  ** 1 patient could not be classified because information regarding tumor size was missing | | | | |
